# S Phase–Coupled E2f1 Destruction Ensures Homeostasis in Proliferating Tissues

**DOI:** 10.1371/journal.pgen.1002831

**Published:** 2012-08-16

**Authors:** Jean M. Davidson, Robert J. Duronio

**Affiliations:** 1 Department of Biology, The University of North Carolina at Chapel Hill, Chapel Hill, North Carolina, United States of America; 2 Lineberger Comprehensive Cancer Center, The University of North Carolina at Chapel Hill, Chapel Hill, North Carolina, United States of America; 3 Program in Molecular Biology and Biotechnology, The University of North Carolina at Chapel Hill, Chapel Hill, North Carolina, United States of America; University of Washington, United States of America

## Abstract

Precise control of cell cycle regulators is critical for normal development and tissue homeostasis. E2F transcription factors are activated during G1 to drive the G1-S transition and are then inhibited during S phase by a variety of mechanisms. Here, we genetically manipulate the single *Drosophila* activator E2F (E2f1) to explore the developmental requirement for S phase–coupled E2F down-regulation. Expression of an E2f1 mutant that is not destroyed during S phase drives cell cycle progression and causes apoptosis. Interestingly, this apoptosis is not exclusively the result of inappropriate cell cycle progression, because a stable E2f1 mutant that cannot function as a transcription factor or drive cell cycle progression also triggers apoptosis. This observation suggests that the inappropriate presence of E2f1 protein during S phase can trigger apoptosis by mechanisms that are independent of E2F acting directly at target genes. The ability of S phase-stabilized E2f1 to trigger apoptosis requires an interaction between E2f1 and the *Drosophila* pRb homolog, Rbf1, and involves induction of the pro-apoptotic gene, *hid*. Simultaneously blocking E2f1 destruction during S phase and inhibiting the induction of apoptosis results in tissue overgrowth and lethality. We propose that inappropriate accumulation of E2f1 protein during S phase triggers the elimination of potentially hyperplastic cells via apoptosis in order to ensure normal development of rapidly proliferating tissues.

## Author Summary

Rapidly growing tissues provide an excellent opportunity to study the careful balance between cell proliferation and apoptosis needed for normal organ structure and function in developing organisms. We present evidence that a transcription factor critical for regulating progression of the *Drosophila melanogaster* cell cycle, E2f1, serves also as an indicator of normal tissue development. E2f1 activation during G1 phase of the cell cycle triggers entry into S phase. E2f1 activity is then rapidly inhibited during S phase by a mechanism that couples E2f1 proteolysis directly to DNA synthesis. Expression during larval development of an S phase-stabilized form of E2f1 results in apoptosis in rapidly proliferating adult wing precursor cells, even when this stabilized E2f1 protein is mutated such that it cannot induce transcription or cell cycle progression. Preventing the ability of S phase-stabilized E2f1 to induce apoptosis results in massive tissue overgrowth. We propose that aberrant E2f1 accumulation during S phase triggers apoptosis in order to remove potentially hyper-proliferative cells and to maintain homeostasis during tissue growth.

## Introduction

During development, cells continually integrate extrinsic and intrinsic signals that control cell growth, proliferation and apoptosis. Mitogenic signals that drive growth and cell proliferation are balanced with apoptotic signals that eliminate damaged or unneeded cells. Genetic changes that inappropriately stimulate cell proliferation, reduce apoptosis, or both disrupt this homeostasis and result in aberrant development or neoplastic diseases like cancer [1]. Understanding the mechanisms that exist to maintain such homeostasis is thus an important area of investigation.

The balance between cell proliferation and cell death in growing tissues must ultimately function through key regulators of the cell cycle. These regulators include the E2F family of transcription factors, which control the expression of many genes responsible for cell proliferation, differentiation and apoptosis [2]. E2Fs are highly conserved proteins that act as either activators or repressors of transcription based on protein partners and structural features. As key mediators of cell proliferation and apoptosis, tight regulation of E2F activity is essential for normal development in mammals, flies, worms, and plants [2], [3]. The best-characterized mode of regulation involves members of the retinoblastoma (pRb) tumor suppressor protein family, which bind to and inhibit those members of the E2F family that dimerize with DP proteins [2]. In addition, pRb family/E2F complexes function as transcriptional repressors [4]. Loss of pRb function causes ectopic proliferation and apoptosis that is partially repressed by reducing E2F activity [5].

pRb family regulation of E2F occurs in quiescent cells and during G1 phase. Several pRb-independent mechanisms have been described that regulate activator E2Fs outside of G1, including Cyclin A/Cdk2-dependent phosphorylation of the DP subunit [6], [7], [8], SCF^Skp2^-directed proteolysis [9], 10, antagonism by the atypical E2F7 and E2F8 proteins [4], [11], [12], and binding to DP-4 [13]. These mechanisms are thought to down-regulate transcriptional activation by E2Fs during S phase or after DNA damage. In particular, disruption of Cyclin A/Cdk2 phosphorylation of E2F1 causes S phase defects and apoptosis in mouse cells, as does simultaneous loss of E2F7 and E2F8 [7], [8], [11]. In addition, E2F7/8 mutation in mice results in lethality, indicating that E2F7/8 play an essential role in the E2F regulatory network during development [11]. Mouse mutant genotypes that would specifically determine the contribution to development of Cyclin A/Cdk2 phosphorylation or the other modes of pRb-independent E2F inhibition have not been developed.

Here we examine the function of pRb-independent E2F regulation in developing *Drosophila* tissues, where E2F regulatory pathways are simpler than in mammals. While eight mammalian E2F genes encode nine distinct proteins (5 activators and 4 repressors), *Drosophila* encodes a single E2F activator (E2f1) and a single E2F repressor (E2f2), both of which bind the single Dp protein [2]. The primary cell cycle regulator is E2f1/Dp, which activates the transcription of replication factor genes and is negatively regulated by Rbf1, one of the two *Drosophila* pRb family members [14]. *E2f1* mutant cells proliferate poorly [15], [16], [17], in part because of E2f2-mediated repression [18], [19]. Conversely, over-expression of E2f1 can drive cells into S phase [20], [21], [22]. E2f1 over-expression also induces apoptosis [17], [20], [21], and this may reflect the positive role E2f1 plays in developmentally controlled and DNA damage induced apoptosis [23], [24], [25], [26]. While many S phase and apoptotic transcriptional targets of E2f1 have been described [27], [28], the aspects of E2f1 regulation that coordinate the expression of these targets in rapidly growing tissues to achieve the proper balance of cell proliferation and apoptosis are not well understood.

In addition to the evolutionarily conserved pRb mode of activator E2F regulation, *Drosophila* E2f1 is inhibited by rapid destruction during early S phase [20], [29], [30], [31]. We recently determined that this S phase destruction is mediated by a “PIP degron" in E2f1 [32]. PIP degrons promote direct binding to DNA-loaded PCNA and the subsequent recruitment of the CRL4^Cdt2^ ubiquitin E3 ligase, thereby coupling proteolysis with DNA synthesis that occurs during S phase or after DNA damage [33], [34]. *Drosophila* E2f1 thus joined a small but growing number of proteins involved in genome duplication and maintenance that are regulated by CRL4^Cdt2^ [33], [34].

We previously demonstrated that expression of an S phase-stabilized E2f1 causes cell cycle acceleration, apoptosis, and developmental defects [32]. Because similar levels of wild type E2f1 expression, which is degraded during S phase, do not induce these phenotypes, we concluded that accumulation of E2f1 during S phase is poorly tolerated during development. However, we did not determine whether apoptosis and the developmental defects were a consequence of changes to the cell cycle in response to hyperactive E2f1 transcriptional activity, or to some other consequence of E2f1 accumulation during S phase. To explore this issue, we used assays in larval imaginal discs to understand the *in vivo* consequences of stabilizing E2f1 during S phase in developing tissues, focusing specifically on which activities of the E2f1 protein (e.g. DNA binding or Rbf1 binding) were responsible for the deleterious phenotypes resulting from stabilization during S phase.

We demonstrate here that the apoptosis and developmental defects caused by accumulation of E2f1 protein during S phase do not require E2f1's ability to induce transcription and cell cycle progression. Instead, apoptosis may occur via alleviation of Rbf1-dependent repression of the pro-apoptotic gene *hid*. We also show that simultaneously stabilizing E2f1 in S phase and blocking apoptosis results in extensive tissue overgrowth. We propose that inappropriate S phase accumulation of E2f1 protein in proliferating *Drosophila* cells triggers a form of proliferative stress, and that the cells experiencing this stress are consequently eliminated via apoptosis in order to prevent hyper-proliferation and maintain homeostasis during rapid tissue growth.

## Results

### An *in vivo* assay for S phase–coupled E2f1 destruction

**Figure 1:**
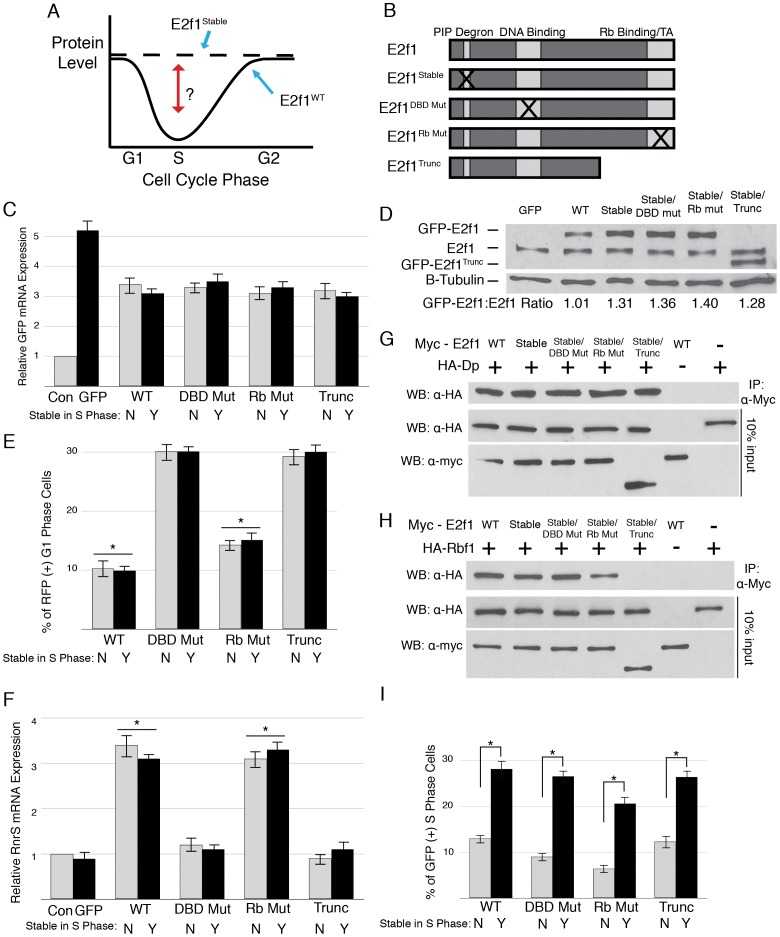
Domain mutations disrupt critical E2f1 functions. A) Schematic of the experimental paradigm. B) Schematic representation of E2f1 alleles used in this study. C) qRT-PCR quantification of GFP-containing mRNA in en-Gal4 wing discs expressing GFP or the indicated GFP-E2f1 fusion proteins that lack (grey; “N") or contain (black; “Y") the PIP-3A mutation (Figure S1A) relative to a non-transgenic *w^1118^* control (Con). Error bars represent the standard error of three independent experiments. These designations will be used throughout the remaining figures. UAS-GFP expression was greater than any E2f1 construct because the UASt promoter was used rather than UASp. D) Anti-E2f1 western blot measuring GFP-E2f1 and endogenous E2f1 expression in third instar imaginal wing discs. The ratio of transgene expression to endogenous E2f1 expression is shown below. E) Quantification by flow cytometry of RFP-positive G1 cells from trypsin-dissociated en-Gal4, UAS-RFP wing discs expressing GFP or the indicated GFP-E2f1 fusion proteins. * p<0.001 as compared to GFP-E2f1 expression. F) qRT-PCR quantification of *RnrS* mRNA in en-Gal4 wing discs expressing GFP or the indicated GFP-E2f1 fusion proteins. G, H) Co-immunoprecipitation analysis of Myc-E2f1 and HA-Dp (G) or HA-Rbf1 (H) from transiently transfected S2 cells. I) Quantification by flow cytometry of GFP-positive S phase cells from trypsin-dissociated en-Gal4 wing discs expressing GFP or the indicated GFP-E2f1 fusion proteins. * p<0.001 compared between stabilized and normally degraded proteins.

In order to examine the biological functions of CRL4^Cdt2^-mediated destruction of E2f1 during tissue growth and development, we examined larval wing imaginal discs, which grow from a ∼50 cell primordium to a ∼50,000 cell epithelial monolayer via canonical G1-S-G2-M cell division cycles and then differentiate into the adult wing during pupal development [17], [35]. Imaginal disc growth is highly tuned to modulate the balance between proliferation and apoptosis in response to particular stimuli. A dramatic example is the ability of wing discs to utilize “compensatory proliferation" in order to achieve normal wing development when as many as 50% of the disc cells have been killed via apoptosis following ionizing radiation [36]. This is possible because *Drosophila* apoptotic cells release mitogens such as Dpp and Wg that signal neighboring cells to begin proliferating and replace the dying cells [37], [38], [39]. We utilized this well characterized, rapidly proliferating tissue to examine the consequences of disrupting the normal S phase-coupled destruction of E2f1 (Figure 1A). We sought to determine the extent to which this destruction contributes to the balance between proliferation and apoptosis.

We previously established an assay for E2f1 destruction during S phase using flow cytometry of cultured *Drosophila* S2 cells expressing GFP-E2f1 fusion proteins [32]. In this assay, a mutation of E2f1 predicted to disrupt interaction with PCNA (GFP-E2f1^PIP-3A^) or a mutation predicted to abrogate CRL4^Cdt2^ binding (GFP-E2f1^R161A^) blocks S phase destruction (Figure S1A, S1B) [40]. We adapted this assay to wing imaginal discs in order to establish a quantifiable assay for measuring E2f1 destruction *in vivo*. We used *engrailed*-Gal4 (en-Gal4) to induce GFP or GFP-E2f1 fusion protein expression (e.g. “en-Gal4>GFP") in all cells of the posterior compartment of the disc (Figure S1C). Wing discs were dissected from third instar larvae, dissociated into individual cells by trypsin digestion, and subjected to flow cytometry after staining cells with a DNA binding dye [41]. We were able to directly compare the cell cycle profile of GFP-expressing posterior compartment cells to GFP-negative, anterior compartment control cells from the same tissue (Figure S1D–S1F). Because GFP is stable throughout the cell cycle, all posterior compartment S phase cells from en-Gal4>GFP discs were also GFP-positive (Figure S1D, S1G). In contrast, en-Gal4>GFP-E2f1 posterior compartment cells with an S phase DNA content were unlikely to be GFP-positive, because GFP-E2f1 is destroyed during S phase (Figure S1E, S1G). Only ∼12% of all GFP-E2f1 expressing cells in the posterior compartment were also in S phase, whereas ∼27% of GFP-expressing cells were in S phase (Figure S1G). This S phase destruction requires an intact PIP degron, as expression of GFP-E2f1^PIP-3A^ resulted in an amount of GFP-positive posterior compartment S phase cells similar to GFP controls (Figure S1F, S1G). (For the rest of this manuscript we will refer to stabilized E2f1^PIP-3A^ as E2f1^Stable^). These data extend our previously published wing disc experiments, in which we measured the effects of E2f1^Stable^ expression on cell cycle progression by flow cytometry, but not directly on E2f1 destruction [32].

We previously showed that E2f1^Stable^ expression accelerates cell cycle progression by using en-Gal4 to drive expression of GFP or GFP+GFP-E2f1 fusion proteins together in the posterior compartment of wing imaginal discs [32]. To measure such cell cycle effects for this study, we switched to co-expressing RFP with GFP or GFP-E2f1 fusion proteins (Figure S1C). By determining the number of RFP-positive cells in each phase of the cell cycle via DNA content, we can obtain a cell cycle profile of all posterior compartment cells. E2f1 stimulates cell cycle progression in wing imaginal disc cells by reducing the duration of G1 phase [17]. Therefore, by comparing the number of RFP-positive cells with G1 DNA content after expression of GFP or GFP-E2f1, we are able to quantify the extent to which E2f1 expression affects the cell cycle. For example, expression of either GFP-E2f1 or GFP-E2f1^Stable^ caused a decrease in the percentage of cells in the population with a G1 DNA content compared to GFP expression alone (∼11% versus ∼28%, respectively; Figure S1H), indicating that both wild type and S phase-stabilized E2f1 proteins are equally able to increase the rate of wing disc cell cycle progression by reducing G1 length, as we previously described [32].

### E2f1 domain mutations disrupt critical E2f1 functions

We previously demonstrated that in addition to an increase in the rate of cell proliferation, ectopic expression of E2f1^Stable^ in wing imaginal discs caused an increase in apoptosis [32]. Interestingly, under the conditions of these experiments, expression of wild type E2f1 did not induce apoptosis although it did increase the rate of proliferation. We therefore hypothesize that E2f1^Stable^ -induced apoptosis is not merely a consequence of increased cell proliferation resulting from excess E2f1 activity, but that the stabilization of E2f1 specifically in S phase triggers cell death.

**Figure 2:**
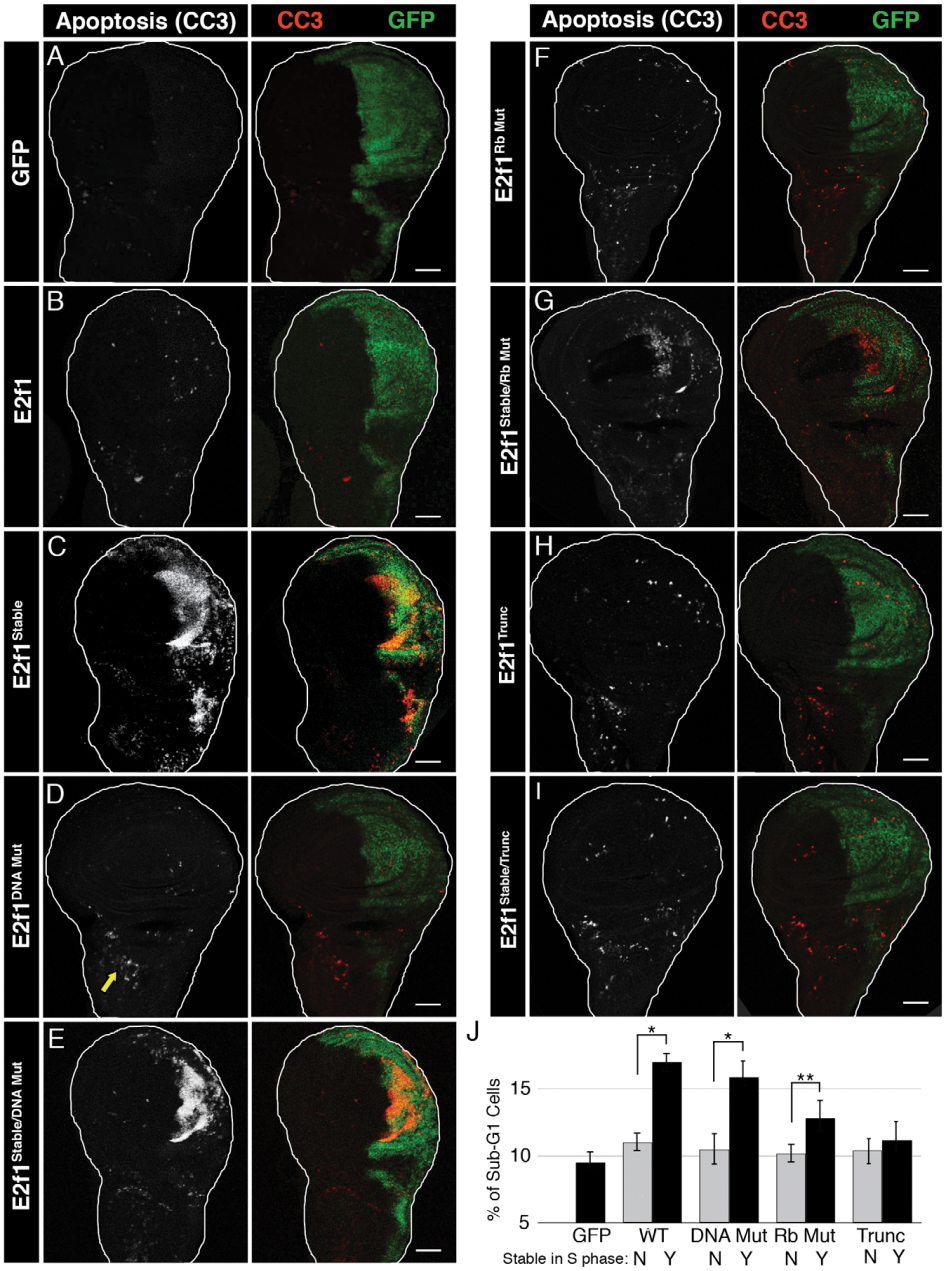
E2f1^Stable^-induced apoptosis requires Rbf1 binding but not DNA binding. A–I) Detection of apoptosis via Cleaved Caspase-3 (CC3, red) staining of third instar larval wing imaginal discs expressing the indicated GFP-E2f1 (GFP, green) proteins with en-Gal4. Arrow in D indicates an example of apoptosis observed in wild type wing discs. Bars = 50 µM. J) Quantification by flow cytometry of GFP-positive apoptotic cells from trypsin-dissociated en-Gal4 wing discs expressing GFP or the indicated GFP-E2f1 fusion proteins. Error bars represent the standard error of three independent experiments. ** p<0.01, * p<0.001.

To explore this phenomenon further, we constructed variant forms of E2f1^Stable^ in which key E2f1 activities–DNA binding, Rbf1 binding, and transactivation–were disrupted in order to determine those aspects of E2f1 function that are necessary for E2f1^Stable^ -induced phenotypes (Figure 1B). To disrupt DNA binding, we mutated to alanines four amino acids in the highly conserved RRXYD motif (R292, R293, Y295 and D296) that make direct contact with bases in the E2F recognition sequence (E2f1^DBD Mut^) [42]. Mutation of the E2F RRXYD motif was previously demonstrated to block DNA binding [43]. To disrupt interaction with Rbf1, we engineered into our constructs a previously characterized missense mutation (L786Q) within the COOH-terminal Rbf1-binding domain of E2f1 that disrupts normal Rbf1-E2f1 interaction but leaves E2f1 transactivation intact (E2f1^Rb Mut^) [44]. Because this single amino acid change does not completely eliminate Rbf1-E2f1 interaction (see Figure 2E), we also engineered into our constructs a previously described mutation (*E2f1^i2^*) that inserts a stop codon at amino acid Q527 [45]. This allele produces a truncated protein lacking the COOH terminal 1/3 of E2f1, thereby eliminating both transactivation function and Rbf1 binding. We will refer to this allele as E2f1^Trunc^.

We first determined whether the mutations we engineered affected GFP-E2f1 and GFP-E2f1^Stable^ activity as predicted. We generated UAS-transgenic lines and selected for analysis those that expressed equivalent amounts of GFP-E2f1 mRNA when driven with en-Gal4 (Figure 1C). Each GFP-E2f1^Stable^ mutant protein accumulated to a similar level that was 30–40% higher than either GFP-E2f1 or endogenous E2f1 (Figure 1D). This increase in protein level is consistent with stabilization only during S phase, which represents about one third of the total cell cycle length (Figure 1F, GFP only).

We next assessed the ability of the E2f1 mutant proteins to drive cell cycle progression and to activate E2f1 target gene expression. The GFP-E2f1^Rb Mut^ and GFP-E2f1^Stable/Rb Mut^ Rbf1 binding mutants with intact transactivation domains were able to promote cell cycle progression (Figure 1E). In contrast, expression of either GFP-E2f1 or GFP-E2f1^Stable^ proteins with mutations that disrupt the transcriptional activity of E2f1, either by blocking DNA binding (GFP-E2f1^DBD Mut^) or removing the transactivation domain (GFP-E2f1^Trunc^), failed to shorten G1 (Figure 1E). Identical results were obtained using S2 cells (Figure S1I).

Mutations that disrupt DNA binding or transactivation should prevent E2f1 from activating expression of replication factor genes. To test this prediction, we measured the level of *RnrS* mRNA, a well-known E2f1-regulated transcript [15]. While expression of GFP did not change the level of *RnrS* mRNA, both GFP-E2f1 and GFP-E2f1^Stable^ expression resulted in a ∼3 fold increase in *RnrS* mRNA in wing imaginal discs (Figure 1F). Similar to the cell cycle progression results, those GFP-E2f1 or GFP-E2f1^Stable^ mutant derivatives that are predicted to be deficient for E2f1 transcriptional activity (GFP-E2f1^DBD Mut^ and GFP-E2f1^Trunc^) were unable to induce *RnrS* expression, while the Rbf1 binding point mutant (GFP-E2f1^Rb Mut^) induced *RnrS* expression similarly to GFP-E2f1 (Figure 1F). Thus, the introduction of the S phase stabilizing mutation did not alter the transcriptional activity of E2f1.

E2f1 requires dimerization with Dp for transcriptional activity and Rbf1 binding for normal regulation in G1 phase [46]. To determine whether our mutations affected Dp or Rbf1 binding, we transiently transfected Myc-E2f1 with either HA-Dp or HA-Rbf1 into S2 cells and performed co-immunoprecipitation assays. All of the E2f1^Stable^ mutant proteins bound Dp equivalently to wild type E2f1 (Figure 1G). Likewise, we found that E2f1, E2f1^Stable^, and E2f1^Stable/DBD Mut^ precipitated similar amounts of Rbf1 (Figure 1H). In contrast, E2f1^Stable/Rb Mut^ precipitated a reduced amount of Rbf1 relative to E2f1, and the truncated E2f1^Stable/Trunc^ showed no ability to precipitate Rbf1 (Figure 1H). These data indicate that we have successfully created PIP degron mutant derivatives of E2f1 that have the predicted effects on the ability to activate transcription and drive cell cycle progression (GFP-E2f1^Stable/DBD Mut^), bind Rbf1 (E2f1^Stable/Rb Mut^), or both (E2f1^Stable/Trunc^).

### E2f1 destruction does not require DNA binding or interaction with Rbf1

We next asked whether any of these mutations affected S phase-coupled E2f1 destruction. Using either the wing disc or S2 cell flow cytometric assays, we found that E2f1^DBD Mut^, E2f1^Rb Mut^, and E2f1^Trunc^ are each destroyed during S phase in a PIP degron-dependent manner (Figure 1I, Figure S1J) [32]. These data indicate that neither the DNA binding, Rbf1 interaction, or transactivation domains of E2f1 are required for S phase-coupled destruction. We previously demonstrated that E2f1 destruction during S phase requires Dp [32], a result that could be interpreted as a requirement for E2f1/Dp DNA binding [34]. However, an alternative interpretation from our observations that the E2f1^DBD Mut^ protein binds Dp and is destroyed normally during S phase is that E2f1/Dp heterodimers are the preferred substrate of CRL4^Cdt2^. In addition, these data suggest that a nuclear pool of E2f1/Dp that is not bound to DNA can interact with PCNA at replication forks and recruit the ubiquitylation machinery.

### Rbf1 binding but not DNA binding is required for S phase–stabilized E2f1 to induce apoptosis

As we showed previously [32], GFP-E2f1^Stable^ induces apoptosis in wing imaginal discs although expression of GFP-E2f1 or GFP does not (Figure 2A–2C). We hypothesized that some activity of E2f1 is necessary to cause cell death only when the protein is inappropriately stabilized in S phase. To determine which functional domains of GFP-E2f1^Stable^ were required to induce apoptosis, we expressed GFP-E2f1^Stable^ variants containing each of the three mutations described above and stained wing imaginal discs with anti-cleaved caspase 3 antibodies (CC3). We first examined the E2f1 DNA binding domain mutant. As expected, GFP-E2f1^DBD Mut^, which does not function as a transcription factor or cell cycle regulator, did not induce apoptosis (Figure 2D). Very surprisingly, however, we detected robust CC3 staining when this protein was stabilized during S phase with the PIP3A mutation (GFP-E2f1^Stable/DBD Mut^) (Figure 2E). This result indicates that apoptosis in response to stabilizing E2f1 in S phase is neither a consequence of aberrant cell cycle progression or E2f1 target gene expression, nor is it solely due to gross over-expression as the normally degradable E2f1^DBD Mut^ did not cause this phenotype.

We next addressed whether GFP-E2f1^Stable^-induced apoptosis requires an interaction with Rbf1. Expression of GFP-E2f1^Rb Mut^ did not induce apoptosis (Figure 2F). The S phase-stabilized Rbf1 binding mutant GFP-E2f1^Stable/Rb Mut^ caused an increase in CC3 staining compared to controls, but less than we observed with either GFP-E2f1^Stable^ or GFP-E2f1^Stable/DBD Mut^ expression (Figure 2G). Intriguingly, this effect suggested that the ability of S phase-stabilized E2f1 to induce apoptosis requires an interaction with Rbf1 but *not* the ability of E2f to bind to E2F response elements at target genes or to shorten G1 phase. To test the role of the E2f1-Rbf1 interaction further, we examined the C-terminally truncated GFP-E2f1^Stable/Trunc^ protein, which is devoid of Rbf1 binding. Neither expression of the GFP-E2f1^Trunc^ nor the GFP-E2f1^Stable/Trunc^ protein resulted in an increase in CC3 staining (Figure 2H, 2I). Importantly, both the GFP-E2f1^Stable/Rb Mut^ and the GFP-E2f1^Stable/Trunc^ proteins were expressed at levels equivalent to the GFP-E2f1^Stable^ and GFP-E2f1^Stable/DBD Mut^ proteins that induce apoptosis (Figure 1D).

To quantify the apoptosis induced by different GFP-E2f1 proteins, we measured the number of cells within a specific range of sub-G1 DNA content via flow cytometry of dissociated wing discs. By this assay, we detected ∼5% apoptotic cells in GFP-expressing control discs, which likely reflects both the normal low levels of apoptosis present in unperturbed discs (e.g. arrow Figure 2D) and the consequences of the extensive trypsinization required for dissociation (Figure 2J). GFP-E2f1 caused only a slight increase in sub-G1 cells relative to GFP controls, as did the transcriptionally inactive GFP-E2f1^DBD Mut^ (Figure 2J). In contrast, and in correspondence with the CC3 staining of intact discs, expression of GFP-E2f1^Stable^ or GFP-E2f1^Stable/DBD Mut^, which lacks a functional DNA binding domain, caused a significant increase in the apoptotic population of cells relative to controls (Figure 2J). The E2f1^Stable/Rb Mut^ Rbf1-binding mutant triggered apoptosis, but less so than GFP-E2f1 proteins with a wild type Rbf1 binding domain, and the GFP-E2f1^Stable/Trunc^ Rbf1-binding deficient mutant did not significantly increase apoptosis above controls (Figure 2J). These data indicate that interaction with Rbf1 is required for S phase-stabilized E2f1 to induce apoptosis. They also suggest that cells have a mechanism to detect aberrant E2f1 protein levels during S phase that is independent of E2f1's role as a transcription factor.

### E2f1^Stable^ causes defects in the first cell cycle after induction of its expression

**Figure 3:**
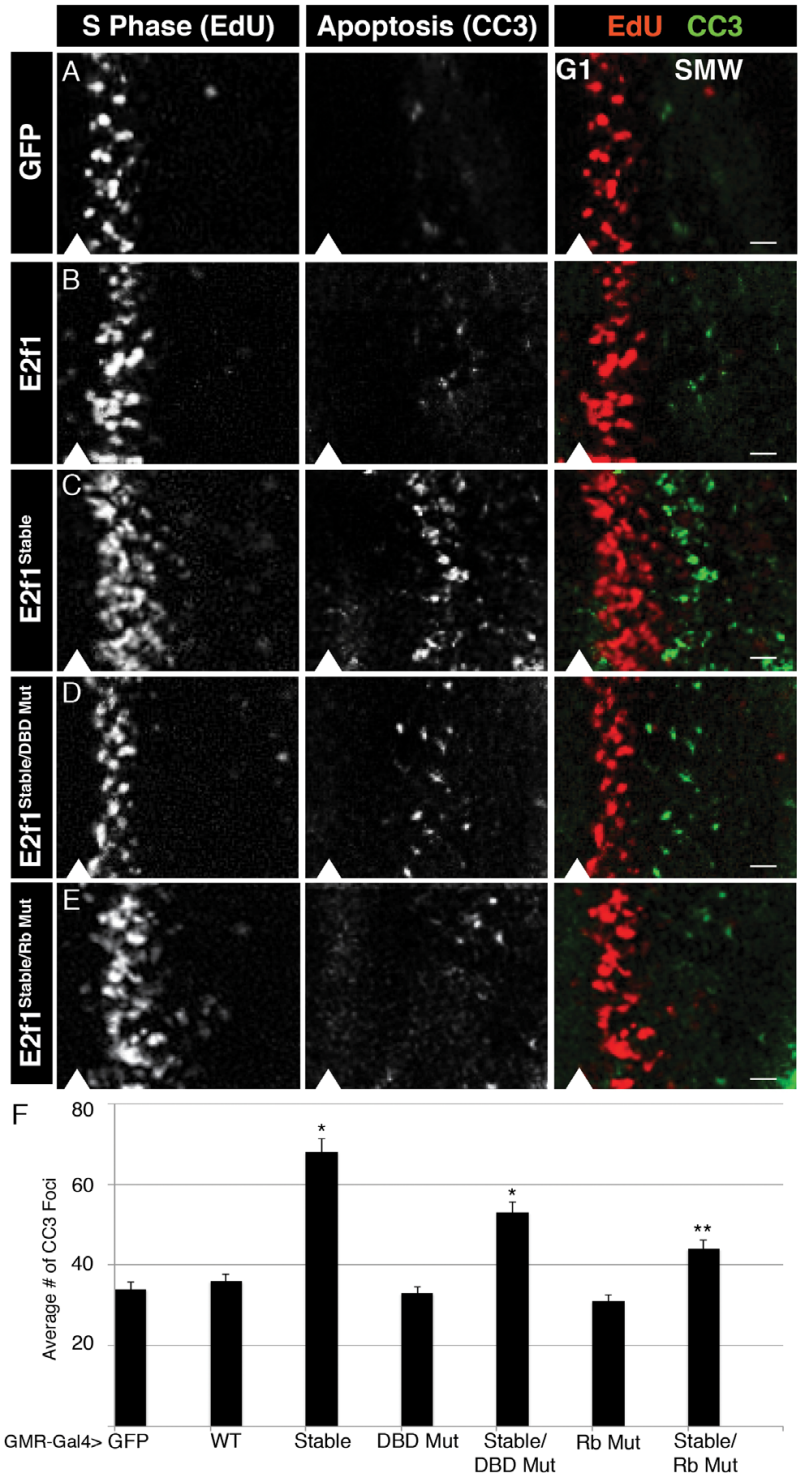
E2f1^Stable^ acts acutely to trigger apoptosis. A–E) Detection of S phase by EdU labeling (red) and apoptosis by CC3 staining (green) in GMR-Gal4 third instar larval eye imaginal discs expressing GFP or the indicated GFP-E2f1 fusion proteins. Arrowheads indicate the position of the MF, with anterior to the left and posterior to the right. Bars = 5 µM. F) Quantification of the number of CC3 positive cells posterior to the MF. * p<0.001 relative to UAS-E2f1 expression.

Our experiments thus far utilize en-Gal4 to drive GFP-E2f1 expression continuously in the posterior compartment during growth of the wing imaginal disc. Because this expression initiates very early during development, we cannot determine whether phenotypes arise in the very first cell cycle after stabilizing E2f1 during S phase, or result from E2f1^Stable^ expression over many cell cycles. To address this issue, we took advantage of the distinct cell cycle program of eye imaginal discs. During third instar larval development, a wave of differentiation associated with a coordinated cell shape change called the morphogenetic furrow (MF) sweeps across the eye disc from posterior to anterior over a period of two days [47]. Cells anterior to the MF are undifferentiated and undergo asynchronous cell proliferation, while cells posterior to the MF differentiate into the neurons and other specialized cell types of the compound eye. Cells within the MF arrest in G1 phase, and as they exit the MF some cells remain in G1 and differentiate while others synchronously reenter a final cell division cycle prior to terminal differentiation called the “second mitotic wave" (SMW) (Figure 3A) [48].

**Figure 4:**
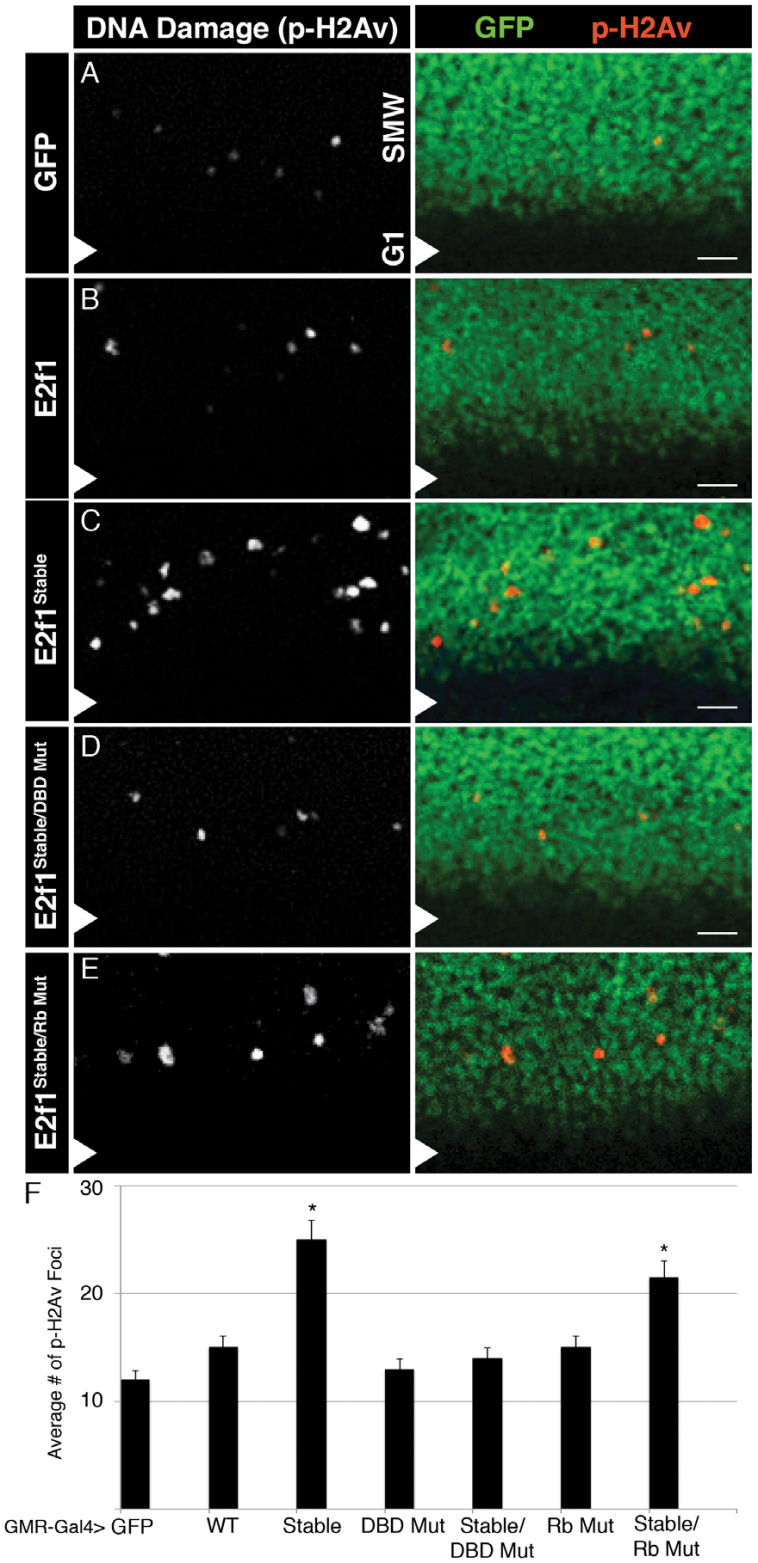
E2f1^Stable^ causes DNA damage. A–E) Detection of DNA damage by anti-phospho-H2Av staining (red) in GMR-Gal4 third instar larval eye imaginal discs expressing GFP or the indicated GFP-E2f1 fusion proteins (green). Arrowheads indicate the position of the MF, with anterior to the bottom and posterior to the top. Bars = 10 µM. F) Quantification of the number of phospho-H2Av positive cells posterior to the MF. * p<0.001 relative to UAS-E2f1 expression.

The GMR-Gal4 driver is activated in late G1 cells of the MF and remains on in all cells posterior to the MF (Figure S2A). By using GMR-Gal4 we could examine the very first cell cycle after expression of the E2f1 transgenes. Normal eye discs have a very organized and stereotypical pattern of S phase in the SMW, and very few cells enter apoptosis immediately posterior to the MF (Figure 3A). Expression of GFP-E2f1 resulted in minimal changes to S phase of the SMW and no significant increase in apoptosis posterior to the MF (Figure 3B, 3F). (Note that others have demonstrated previously that co-expression of E2f1 and Dp with GMR results in ectopic S phase in the MF and apoptosis [21].) In contrast, expression of E2f1^Stable^ disrupted the normal S phase pattern: we observed an increase in the number of cells entering S phase as well as an expansion of the zone of EdU labeling posterior to the MF, suggesting an increase in the length of S phase (Figure 3C). The changes in the S phase pattern caused by GFP-E2f1^Stable^ were accompanied by an increase in DNA damage, as measured by anti-phospho-H2Av staining (Figure 4A–4C, 4F), and apoptosis posterior to the MF (Figure 3C, 3F). There was no change in the number of cells entering mitosis posterior to the MF, as measured by anti-phospho-histone H3 staining (Figure S2B), suggesting that cells die before entering mitosis. In addition, E2f1^Stable^ did not induce apoptosis when expressed in G1-arrested epidermal cells in the embryo (Figure S3), suggesting that apoptosis may be S phase specific. These data suggest that the presence of stabilized E2f1 in even a single S phase can disrupt cell cycle progression, induce DNA damage, and result in apoptosis. Importantly, however, DNA damage and apoptosis does not occur in all of the cells expressing E2f1^Stable^, much like we observed by flow cytometry in the wing discs (Figure 2J).

### E2f1^Stable^ induces apoptosis in two ways in eye discs

We next asked whether the DNA damage and apoptosis observed after S phase stabilization of E2f1 resulted from aberrant cell cycle progression. Expression of GFP-E2f1^Stable/DBD Mut^ did not perturb the organization of S phase in the SMW (Figure 3D) or result in an increased number of phospho-H2Av foci (Figure 4D, 4F), likely because this protein does not alter cell cycle progression. Thus, the DNA damage observed with E2f1^Stable^ was most likely due to proliferation defects, because mutants that failed to shorten G1 did not induce phospho-H2Av. On the other hand, when compared to controls, GFP-E2f1^Stable/DBD Mut^ expression did not cause an increase in phospho-H2Av foci (Figure 4D, 4F), although it still resulted in an increase in apoptosis posterior to the MF Figure 3D, 3F). These data suggest that stabilizing E2f1 in S phase can trigger apoptosis independently of cell cycle effects. The level of apoptosis in GMR>GFP-E2f1^Stable/DBD Mut^ discs was somewhat less than in GMR>GFP-E2f1^Stable^ discs, suggesting a contribution from proliferative stress that is dependent on E2f1 DNA binding (Figure 3F). As in wing discs, apoptosis required an interaction with Rbf1 because GFP-E2f1^Stable/Rb Mut^ expression resulted in reduced apoptosis compared to GFP-E2f1^Stable^ (Figure 3E, 3F). Taken together, these data suggest that two factors contribute to apoptosis when E2f1 is stabilized in S phase in the SMW: proliferative stress caused by aberrant E2f1 activity that leads to DNA damage, and a mechanism independent of E2f1 DNA binding activity that relies on interaction with Rbf1.

We previously reported that E2f1^Stable^ expression in the posterior compartment of the wing discs did not increase the amount of detectable DNA damage [32]. Our eye discs results prompted us to reexamine this issue. Using a different source of anti-phospho-H2Av antibody, we detected an increase in phospho-H2Av foci in wing imaginal discs following expression of GFP-E2f1^Stable^, and as in the eye discs this amount was more than with GFP-E2f1 expression (Figure S4).

### Apoptosis requires full-length E2f1^Stable^

**Figure 5:**
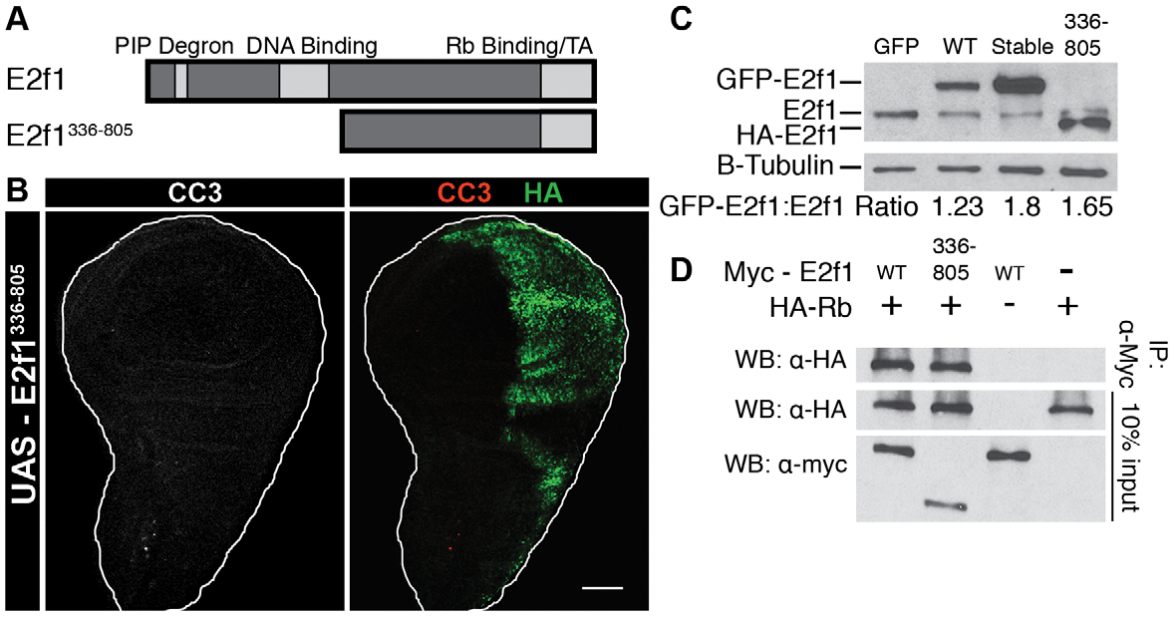
Induction of Apoptosis requires full-length E2f1^Stable^. A) Schematic of the E2f1^336–805^ mutant protein, which contains an NH_2_-terminal HA tag. B) Detection of apoptosis via Cleaved Caspase-3 (CC3, red) staining of third instar larval wing imaginal discs expressing HA-E2f1^336–805^ (anti-HA, green) with en-Gal4. Bar = 50 µm. C) Anti-E2f1 western blot of third instar imaginal wing discs expressing GFP-E2f1, GFP-E2f1^Stable^, or HA-E2f1^336–805^. D) Co-immunoprecipitation analysis of Myc-E2f1 and HA-Rbf1 from transiently transfected S2 cells.

Our data are consistent with the hypothesis that an interaction between S phase-stabilized E2f1 and Rbf1 triggers apoptosis, even when E2f1^Stable^ cannot bind DNA and is functionally inactive as a transcription factor. This result suggests that cells can specifically detect and respond to E2f1/Rbf1 complexes that inappropriately assemble in S phase. However, another possibility is that over-expression of any Rbf1 binding protein would trigger apoptosis. To distinguish between these possibilities, we utilized a NH_2_-terminally truncated allele of E2f1 (E2f1^336–805^) that we previously characterized [49]. E2f1^336–805^ contains only the C-terminal half of the E2f1 protein, and thus lacks the PIP degron and DNA binding domain but retains the Rbf1 binding and transactivation domains (Figure 5A). We hypothesized that this protein would interact with Rbf1 during S phase, but not trigger apoptosis because of the absence of a domain necessary for cells to detect the E2f1^Stable^/Rbf1 complex. Indeed, en-Gal4 expression of E2f1^336–805^ failed to induce apoptosis (Figure 5B), even though this protein accumulated to levels similar to GFP-E2f1^Stable^ (Figure 5C) and efficiently interacted with Rbf1 in co-immunoprecipitation assays (Figure 5D). These data indicate that interaction with Rbf1 is not by itself sufficient to induce apoptosis, and suggest that full-length E2f1^Stable^ is specifically recognized by cells to induce apoptosis.

### Stabilizing E2f1 during S phase causes apoptosis by inducing *hid* expression

What mechanism could explain the induction of apoptosis upon stabilization of a transcriptionally inert, but Rbf1 binding-proficient, E2f1 protein during S phase? Recent work from several laboratories showed that loss of Rbf1 function causes apoptosis in several developmental contexts by triggering expression of the pro-apoptotic gene, *hid* [18], [24], [25], [50], [51]. Hid is homologous to SMAC/Diablo family proteins that function to antagonize IAPs, which act to block activator caspases. *hid* expression triggers an apoptotic cascade by antagonizing DIAP1, thus releasing inhibition of the initiator caspase Dronc and activating the effector caspase Drice [36], [52].

**Figure 6:**
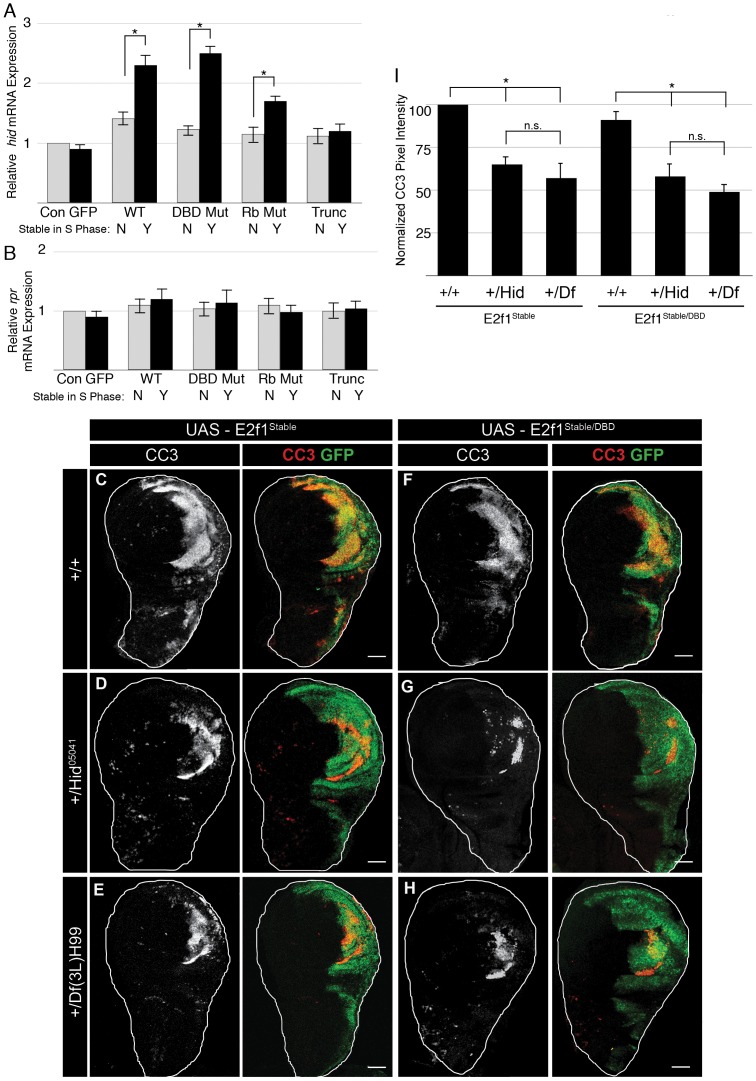
Stabilizing E2f1 during S phase induces *hid* expression. A, B) qRT-PCR quantification of *hid* mRNA (A) or *rpr* mRNA (B) in en-Gal4 wing discs expressing GFP or the indicated GFP-E2f1 fusion proteins that lack (grey) or contain (black) the PIP3A mutation relative to a non-transgenic *w^1118^* control (Con). * p<0.001. C–I) Detection of apoptosis via Cleaved Caspase-3 (CC3, red) staining of third instar larval wing imaginal discs expressing the indicated GFP-E2f1 (GFP, green) proteins with en-Gal4. En-Gal4>E2f1^Stable^ (C–E) or E2f1^Stable/DBD^ (F–H) in either a wildtype *hid* background (+/+), or heterozygous *Hid^05141^*/+ or *Df(3L)H99*/+ backgrounds. I) Quantification of CC3 pixel intensity as measured using ImageJ. All genotypes were normalized against E2f1^Stable^; +/+ cleaved caspase-3 levels. * p<0.001. n.s. not significant. n = 12 discs for each genotype.

We hypothesized that GFP-E2f1^Stable^ or GFP-E2f1^Stable/DBD Mut^ binds to Rbf1 and disrupts its function, resulting in activation of *hid* expression. This hypothesis predicts that GFP-E2f1^Stable^ or GFP-E2f1^Stable/DBD Mut^ expression will increase *hid* expression, while E2f1 mutants that cannot bind Rbf1 will fail to increase expression. To test this prediction, we used qRT-PCR to measure the levels of *hid* mRNA in wing imaginal discs expressing the various GFP-E2f1 transgenes with en-Gal4. Consistent with our hypothesis, there was a two-fold increase in *hid* mRNA in GFP-E2f1^Stable^- or GFP-E2f1^Stable/DBD Mut^ -expressing discs relative to those expressing GFP-E2f1 or GFP-E2f1^DBD Mut^ (Figure 6A). Similar levels of *hid* induction were previously observed following ionizing radiation treatments that trigger apoptosis [24]. *hid* expression was not significantly increased by the GFP-E2f1 mutants lacking normal Rbf1 binding activity (Figure 6A). To test whether the *hid* de-repression was a specific response to stabilizing E2f1 in S phase, we measured expression of another pro-apoptotic gene, *reaper*, which is not de-repressed by *Rbf1* mutation [24]. We detected no increase in *reaper* mRNA in discs expressing any GFP-E2f1 transgene (Figure 6B).

To test if *hid* expression contributed to E2f1^Stable^-induced apoptosis, we determined whether reducing *hid* gene dose would result in a decrease in apoptosis. We utilized two different *hid* alleles: *hid^05014^*, containing a transposable element insertion between amino acids 105 and 106 in the open reading frame that effectively reduces *hid* expression [53], and *Df(3L)H99*, which deletes the entire *hid* gene as well as the neighboring pro-apoptotic genes, *reaper* and *grim* [53], [54]. Wing discs heterozygous for either *hid* allele contained a significantly reduced amount of apoptosis after GFP-E2f1^Stable^ or GFP-E2f1^Stable/DBD^ expression compared to controls (Figure 6C–6H, 6I). Quantification of CC3 staining revealed no significant difference between the results obtained with *hid^05014^* (Figure 6D, 6G, 6I) and *Df(3L)H99* (Figure 6E, 6H, 6I). This result suggests that *grim* and *reaper* do not contribute as substantially as *hid* to E2f1^Stable^-induced apoptosis, consistent with our failure to detect an increase in *reaper* expression by E2f1^Stable^ and its derivatives (Figure 6B). These data support the idea that stabilizing E2f1 during S phase results in disruption of Rbf1 function leading to de-repression of *hid* expression and apoptosis.

### E2f1^Stable^ induces hypertrophy when cells are prevented from executing apoptosis

Why would *Drosophila* cells induce a potent activator of apoptosis in response to elevated E2f1 protein levels during S phase? We considered the possibility that a small number of individual cells in a growing population of adult precursor cells, like those in wing imaginal discs, might stochastically experience hyper-expression of E2F that would manifest as the presence of excess E2f1 protein in S phase. Such cells would be eliminated by apoptosis, thereby helping to maintain growth homeostasis by suppressing the appearance of potentially hyperplastic cells that could lead to aberrant overgrowth. If this was a developmentally important event, then blocking the ability of tissues to eliminate such cells by apoptosis should disrupt normal development.

**Figure 7:**
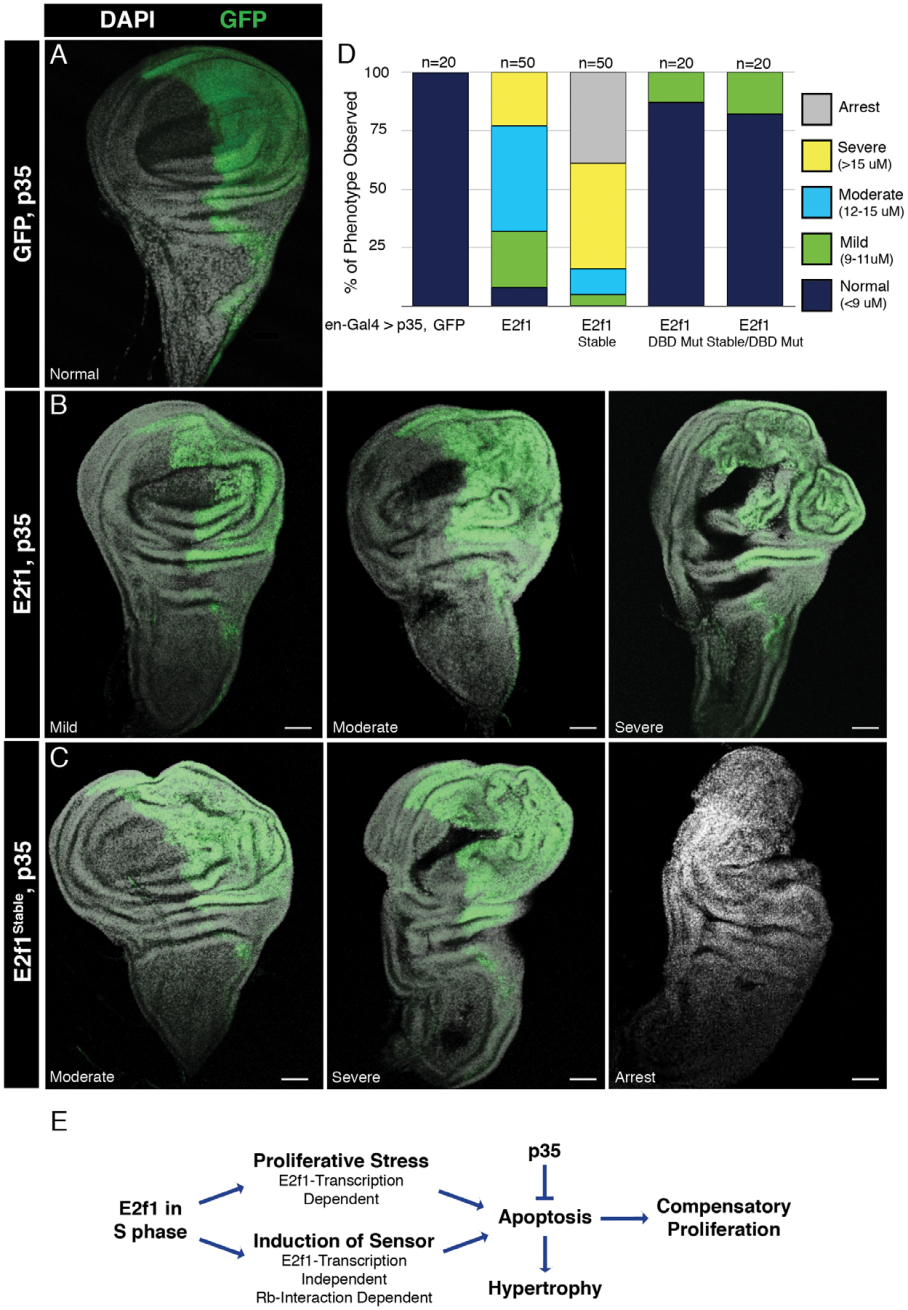
E2f1^Stable^ causes homeostasis defects and tissue hyperplasia. A–C) Detection of GFP or the indicated GFP-E2f1 proteins (green) in en-Gal4, UAS-p35 wing discs stained with DAPI (white). Scale bars indicate 50 µm. D) Quantification of morphological defects by microscopically measuring the thickness of the posterior compartment of the indicated en-Gal4>GFP-E2f1 wing discs. Measurements were obtained by counting the number of 1 micron sections required to visualize all the way through the posterior compartment of the tissue. Bars = 50 µM. E) E2f1^Stable^ induces apoptosis in two ways.

To test this idea, we used en-Gal4 to co-express GFP-E2f1 transgenes in wing imaginal discs together with baculovirus p35, which efficiently blocks apoptosis in *Drosophila* cells [55]. Expressing p35 together with GFP had no deleterious effects on wing disc development (Figure 7A). In contrast, GFP-E2f1/p35 co-expression resulted in a range of morphological defects caused by hyperplastic growth. While some GFP-E2f1/p35-expressing discs appeared normal, most displayed various degrees of overgrowth in the posterior portion of the disc (Figure 7B). We quantified this overgrowth by microscopically measuring posterior compartment “thickness", which we defined as the sum of the number of confocal sections one micron apart required to image through the entire posterior compartment. Using this measurement we binned the discs into four phenotypic categories: normal (<9 µm), mild (9–11 µm), moderate (12–15 µm), and severe (>15 µm) (Figure 7D). GFP-E2f1^Stable^/p35 expression caused a more severe phenotype than did GFP-E2f1/p35 expression. None of the discs were normal, and a larger percentage of the discs fell into the severe overgrowth category (Figure 7C, 7D). In addition, GFP-E2f1^Stable^/p35 expression caused the appearance of a unique fifth phenotype in ∼1/3 of the discs, which we called “arrest" (Figure 7C, 7D). In these discs the posterior compartment was almost absent, as confirmed by co-expression of RFP. We speculate on the origin of this class of discs in the Discussion. Expression of p35 together with either GFP-E2f1 or GFP-E2f1^Stable^ caused 100% lethality. Importantly, the hyperplastic growth induced by GFP-E2f1 or GFP-E2f1^Stable^ required the normal transcriptional and cell cycle-promoting activity of E2f1, as co-expression of p35 with GFP-E2f1^DBD Mut^ or GFP-E2f1^Stable/DBD Mut^ resulted primarily in normal wing discs and did not cause lethality (Figure 7D). These data indicate that the developmental effects of E2f1 hyper-activity during tissue growth are exacerbated by simultaneously blocking apoptosis and E2f1 destruction in S phase.

## Discussion

We show here that stabilizing the single *Drosophila* activator E2f1 in S phase results in apoptosis that is necessary to prevent hypertrophy of wing imaginal discs. We conclude from these data that hyper-accumulation of E2f1 during S phase represents a form of proliferative stress during development that is sensed by the apoptotic machinery and results in the elimination of cells with excess E2f1 activity to maintain homeostasis during tissue growth.

### S phase–coupled E2f1 destruction helps balance cell proliferation with apoptosis

What might be the function of a *Drosophila* cell's ability to detect abnormal accumulation of E2f1 protein during S phase and subsequently trigger apoptosis? One possibility is that accumulation of E2f1 during S phase resembles instances of abnormally high E2f1 activity that might occur sporadically during rapid growth of a population of precursor cells such as those in the wing imaginal disc. These events could be caused by stochastic or even genetic changes that affect either *E2f1* gene transcription or the ability of the CRL4^Cdt2^/PCNA pathway to destroy E2f1 after replication factor genes are activated in late G1. The cell's ability to detect E2f1 accumulation in S phase clears these potentially hyperplastic cells from developing tissues via apoptosis, consequently contributing to the balance between cell proliferation and cell death that is necessary for normal tissue growth.

Growing *Drosophila* imaginal discs possess another mechanism of homeostasis in which a process of compensatory proliferation is activated in order to achieve normal tissue development when as many as 50% of cells are killed by external stimuli like radiation-induced DNA damage [56]. Indeed, in spite of high levels of apoptosis (15% of the cells), 50% of en-Gal4>E2f1^Stable^ progeny survive until adulthood with about 2/3 of these surviving flies containing wings with somewhat mild morphological defects [32]. Blocking apoptosis with baculovirus p35 when E2f1^Stable^ is expressed shifts the cell proliferation/apoptosis balance too strongly in favor of cell proliferation, resulting in massive hypertrophy and 100% lethality.

p35 is a broad caspase inhibitor that blocks effector caspase activity at a step downstream of their proteolytic activation [55]. Therefore, cells expressing p35 can initiate apoptosis, but lack the capacity to complete it and are referred to as “undead cells." These undead cells produce signals that stimulate unaffected neighboring cells to proliferate [36]. Thus, the dramatic hypertrophy we see in E2f1^Stable^/p35 wing discs might be the result of two synergizing growth signals: hyper-active E2f1 and compensatory proliferation from undead cells. Our experiments cannot precisely discern the relative contribution of these two inputs, but E2f1 activity appears to make a larger contribution because E2f1^Stable/DBD Mut^ expression does not cause dramatic overgrowth.

What might explain the 32% of en-Gal4>E2f1^Stable^ discs that displayed a reduced posterior compartment rather than an overgrown one: the “arrest" phenotype in Figure 7? The DNA damage we observed in our eye discs experiments provides a possible answer. Perhaps early in development the “arrest" class of wing discs sustained enough genomic damage to prevent proliferation, resulting in too small a pool of cells that could respond to the hyper-active E2f1 and undead cell signals to support disc overgrowth. Thus, the wide range of phenotypes that we observed in E2f1^Stable^/p35 wing discs may result from multiple influences that act stochastically within the population (Figure 7E).

### A cellular sensor of E2f1 hyper-accumulation

Because endogenous E2f1 is quantitatively destroyed only in S phase, the relative amount of hyper-accumulation of E2f1^Stable^ is greater during S phase than during any other stage of the cell cycle. Therefore, one possibility is that E2f1^Stable^-induced phenotypes result from the stability of E2f1 protein in S phase, and not from general over-expression throughout the cell cycle. Our failure to detect E2f1^Stable^ induced apoptosis in G1-arrested embryonic cells is consistent with this possibility. However, another difference between these embryonic cells and wing discs cells is that the former are cell cycle arrested and the latter are continuingly proliferating during larval development. Thus, another possibility is that S phase-destruction of E2f1 modulates the levels of E2f1 in proliferating cells, and cells that fail to destroy E2f1 during S phase have an increased chance of activating apoptosis at any point in the cell cycle. In either model, S phase E2f1 destruction is not essential for proliferation per se. In marked contrast, E2f1^Stable^ expression blocks endocycle progression [57], suggesting that knocking in E2f1^Stable^ to the endogenous locus would be lethal during development, perhaps even dominant lethal.

E2f1^Stable^ induces apoptosis at least in part through expression of the pro-apoptotic gene *hid*. Surprisingly, these events still occur after expression of an E2f1^Stable^ variant that cannot bind DNA and therefore lacks the ability to stimulate transcription of replication factor genes or cell cycle progression. Instead, E2f1^Stable^ requires the ability to bind Rbf1 to induce *hid* gene expression and apoptosis. Genetic disruption of Rbf1 is well known to result in increased *hid* expression [18], [25], [51]. We therefore propose that the inappropriate accumulation of E2f1 in S phase disrupts some aspect of Rbf1 function leading to *hid* expression and apoptosis.

Our data do not discern either the function of Rbf1 that is disrupted by E2f1^Stable^ or the mechanism of *hid* induction. While the mechanism connecting Rbf1/E2f1 function and *hid* may be indirect, some studies suggest that Rbf1 and/or E2f1 could regulate *hid* directly. Su and colleagues recently demonstrated that *Drosophila* wing disc cells undergo apoptosis in response to ionizing radiation independently of p53 and that this response requires E2f1 and is triggered by *hid* expression [26]. In eye discs, loss of Rbf1 function in the MF results in apoptosis that requires E2f1 transactivation function and is accompanied by *hid* expression [18], [50]. However, whether these effects represent a direct induction of *hid* by E2f1 is not clear. E2f1 binding at the *hid* locus has been observed, but the binding site is located ∼1.4 kb upstream of the of the start of *hid* transcription, which is more distal than in well characterized E2F-regulated promoters [58]. When located this far upstream the *hid* E2f1 binding site fails to activate gene expression in S2 cell reporter assays [25]. *hid* is also a target of p53 [59], and so any DNA damage resulting from stabilizing E2f1 during S phase, as we observed in eye discs, may also contribute to the activation of *hid* expression via p53-mediated DNA damage response pathways.

Another possibility is that E2f1, in combination with Rbf1, plays primarily a repressive role at the *hid* locus. In this model, our result that E2f1^Stable^ or E2f1^Stable/DBD Mut^ both induce apoptosis would be explained by disruption of Rbf1/E2f1 repressive complexes at the *hid* locus causing de-repression of *hid* expression. This model has interesting caveats: what protects the Rbf1/E2f1 complex at the *hid* locus from being disrupted by Cyclin E/Cdk2, which is active during S phase and inactivates Rbf1-mediated repression of E2f1 [60], or by CRL4^Cdt2^-mediate destruction of E2f1? Recent data indicate that the dREAM/MMB complex is required for the stability of E2F/Rbf1 repressive complexes in S phase, and acts to protect these complexes from CDK-mediated phosphorylation at non-cell cycle-regulated genes [61]. While there is yet no evidence that dREAM/MMB regulates *hid* [62], this work provides precedent for gene specific Rbf1 regulation during S phase.

Finally, while *hid* might be a critical player in the response to E2f1^Stable^, there are likely other mechanisms responsible for sensing and modulating the apoptotic response to E2f1 levels. For instance, Frolov and colleagues recently demonstrated that a micro-RNA, mir-11, which is located within the last intron of the *Drosophila* E2f1 gene, acts to dampen expression of pro-apoptotic E2f1 target genes following DNA damage [28]. In this way, the normal controls of *E2f1* gene expression modulate apoptosis. In addition, our transgenic constructs lack the normal *E2f1* 3′ UTR, which serves as a site for suppression of E2f1 expression by pumilio translational repressor complexes [63]. Thus, we have bypassed several modes of E2f1 regulation via transgenic expression of E2f1^Stable^.

### Conservation of E2F regulation via different molecular mechanisms

Our finding that stabilized *Drosophila* E2f1 can induce apoptosis independently of transcription and cell cycle progression parallels previous observations made in mammalian cells, albeit with important differences. In mammalian cells, E2F1 can induce apoptosis independently of transcription and cell cycle progression, but apoptosis required E2F1 DNA binding activity, unlike in our experiments [64], [65]. These studies suggested that DNA binding by E2F1 prevented pro-apoptotic promoters from binding repressor E2F family members.

This comparison of results highlights the way similar phenotypic outcomes in different species can arise from different mechanisms. While mammalian activator E2Fs are also inhibited during S phase, they are not subject to CRL4^Cdt2^-mediated, S phase-coupled destruction like *Drosophila* E2f1. Instead, mammalian activator E2Fs are inhibited by direct Cyclin A/Cdk2 phosphorylation [6], [7], [8], targeted for destruction by SCF^Skp2^ [9], [10], and functionally antagonized by E2F7 and E2F8 [11], . The regulation provided by E2F7 and E2F8 is of particular note, as it is essential for mouse development [11]. These atypical E2Fs homo and hetero-dimerize and act redundantly to repress E2F1 target genes independently of pRb family proteins, thus blocking E2F1 from inducing apoptosis [11]. Moreover, the E2F7 and E2F8 genes are E2F1 targets [11], consequently creating a negative feedback loop that limits E2F1 activity after the G1/S transition. A similar negative feedback loop among factors that regulate G1/S transcription exists in yeast [66]. The analogous *Drosophila* negative feedback loop involves E2f1 inducing its own destruction by stimulating *Cyclin E* transcription, which triggers S phase [60]. Therefore, the evolution of eukaryotes has resulted in the use of different molecular mechanism to achieve negative feedback regulation of G1/S-regulated transcription, and in the case of activator E2Fs this regulation is essential for normal development.

## Materials and Methods

### Molecular biology

E2f1 constructs were generated and expressed using pENTR TOPO (Invitrogen) and Gateway-compatible P element vectors (http://www.ciwemb.edu/labs/murphy/Gateway%20vectors.html).

### Cell culture and transfection

For S phase-coupled protein destruction analysis, S2 cells stably transfected with *hsp70* constructs were heat shocked for 30 minutes at 37°C, which results in GFP or GFP-E2f1 expression in all cells of the population, and allowed to recover at room temperature for 200 minutes prior to analysis by flow cytometry. During the 200 min chase period GFP-E2f1 is destroyed in S phase cells while GFP is not, as measured by the percentage of GFP(+) cells in each phase of the cell cycle. For cell cycle analysis, S2 cells were transfected with plasmid DNA expressing GFP or GFP-E2f1 encoding mRNA from the *Actin5C* promoter and analyzed by flow cytometry 48 hours later.

### Flow cytometry

For flow analysis of wing imaginal discs, at least 15 third instar larvae of the appropriate genotype were dissected in PBS. 30 imaginal discs were collected and immediately dissociated in PBS containing 0.05% Trypsin- EDTA (Gibco), and 1X Hoechst 33342 DNA binding dye (Sigma) and rocked for 3 hours at room temperature. The dissociated tissue was then immediately analyzed using a LSRII Flow Cytometer and Diva software (Becton Dickinson). Cell cycle profiles were calculated using FlowJo™ Software. Percentages of G1, S, and G2 cells were calculated using Modfit LT software (Verity Software House). P values for all experiments were calculated by student's T test.

S2 cells stained with propidium iodide were analyzed by flow analysis as previously described [32] using the Cyan flow cytometer with Summit 4.3 software (Deko).

### qRT–PCR

Total RNA was extracted from 30 third instar wing imaginal discs using Trizol reagent (Invitrogen) and tissue was sheared with eight passes through a 25-gauge needle. 0.75 µg of total RNA was used for reverse transcription with RevertAid Reverse transcription kit (Fermentas). The resulting cDNA was used for qRT-PCR performed using an ABI prism 7700 Sequence Detection system. Relative levels of specific mRNAs were determined by detection of Maxima Sybr Green (Fermentas). Primers are listed in Table 1. Comparative CT methods were used to quantify levels versus control Rp49 mRNA using the manufacturer's protocol.

**Table 1:** Primers for qRT–PCR.

Rp49-Forward:	TACAGGCCCAAGATCGTGAAG
Rp49-Reverse:	GACGCACTCTGTTGTCGATACC
GFP-Forward:	GGAGTACAACTACAACAGCC
GFP-Reverse:	CTTGTACAGCTCGTCCATGCCG
HID-Forward:	CATCAGTCAGCAGCGACAGG
HID-Reverse:	ACGAAAACGGTCACAACAGTTG
Reaper-Forward:	CCAGTTGTGTAATTCCGAACGA
Reaper-Reverse:	GGATCTGCTGCTCCTTCTGC
RnrS-Forward:	CATCTGCCAGATGTCGTGGTAC
RnrS-Reverse:	GAAGTCCGTAACCCCCTTCG

### *Drosophila* genetics and cytology

Transgenic flies were generated by injecting UASp-E2f1 plasmids into *w^1118^* (Best Gene *Drosophila* Injection Services, Chino Hills, CA). UAS-GFP, Engrailed-Gal4, UAS-RFP and UAS-p35 stocks were obtained from the Bloomington Stock Center. For antibody staining, imaginal discs were dissected from third instar larvae in PBS, fixed in 6% paraformaldehyde for 20 minutes at room temperature, then permeabilized for 20 minutes in PBS-1.0% Triton-X. Wing discs were incubated overnight with mouse anti-GFP (1∶1000, Abcam) and rabbit anti-cleaved Caspase-3 (Asp175) (1∶200, Cell Signaling Technology) at 4°C. Secondary antibodies were goat anti-mouse Oregon Green 488 (1∶2000 Invitrogen) and goat anti-rabbit Rhodamine (1∶2000 Invitrogen) for 1 hour at room temperature. Eye discs were dissected, incubated in 10 µg/mL EdU (Click-iT™ EdU Alexa Fluor 594, Invitrogen) for 30 minutes, fixed and permeabilized as described above. EdU was detected according to manufacture protocol. To detect mitosis, eye discs were incubated overnight at 4°C with rabbit anti-PH3 (1∶1000, Abcam) and then with goat anti-rabbit Rhodamine (1∶1000 Invitrogen) for 1 hour at room temperature. For DNA damage detection, rabbit anti-p-H2Av antibody from Kim McKim's lab was incubated over night at 4°C at 1∶1000 and then goat anti-rabbit Rhodamine (1∶1000 Invitrogen). DAPI was added for DNA detection (1∶1000 Invitrogen) for 2 minutes. Tissue samples were analyzed with a Zeiss LSM 510 scanning confocal microscope. Quantification of CC3 and p-H2Av foci was collected by projecting confocal images that were one micron apart through the eye disc of 6 discs per genotype and using ImageJ software to count all foci above threshold detection posterior to morphogenetic furrow. 7 images per disc projected for p-H2Av, 6 images per disc for CC3. Graph shown represents the average number of foci of those 6 discs.

### Western blot and co-immunoprecipitation

30 third instar larvae wing imaginal discs were dissected in PBS then dissociated by eight passes through a 25 gauge needle after addition of ice-cold NP40 buffer with protease inhibitors aprotinin (1∶1000), leupeptin (1∶1000) and PMSF (1∶100). E2f1 protein levels were measured with affinity-purified rabbit anti-E2f1 raised against full-length *Drosophila* E2f1 (1∶1000) [32] overnight at 4°C and anti-rabbit HRP secondary (1∶10,000 GE Healthcare) for 1 hour at room temperature. B-tubulin was used as loading control (1∶1000, Abcam) with anti-rabbit HRP secondary (1∶10,000 GE Healthcare). Co-immunoprecipitation was performed by co-transfecting S2 cells with 2 µg Myc-E2f1 and 1 µg HA-Dp or HA-Rbf1 using the Amaxa transfection system (Lonza) and incubating the cells for 24 hours at 28°C. S2 cells were lysed on ice using NP40 buffer with the protease inhibitor cocktail described above. 10% of each total extract was subjected to western blot analysis with mouse anti-Myc (1∶2000 UNC Hybridoma) or mouse anti-HA (1∶2000, UNC Hybridoma). Secondary antibodies were ECL donkey anti-mouse HRP (1∶10,000, GE Healthcare) and ECL donkey anti-rabbit HRP (1∶10,000, GE Healthcare). The remainder of the extract was incubated overnight at 4°C with 0.5 µg mouse anti-Myc antibody (UNC Hybridoma) and 1/10 volume Protein G Sepharose 4 Fast-Flow beads (GE Healthcare).

## Supporting Information

Figure S1 An *in vivo* assay for S phase-coupled E2f1 destruction. A) Alignment of PIP degrons from known CLR4^Cdt2^ substrates. Amino acids of the PIP box are bold and those of Cdt1 that interact with Cdt2 are underlined. E2f1 contains a PIP box located at amino acids 150–157. E2f1 also contains a basic Arg residue (R161) four amino acids downstream of the PIP box, much like the basic K+4 residue found in the Cdt1 PIP degron. Amino acid changes in E2f1^PIP-3A^ and E2f1^R161A^ mutants, which contain nonfunctional PIP degrons, are shown at the bottom. B) An S2 cell flow cytometry assay to quantify the number of GFP-positive cells that are in S phase. The graph indicates the percentage of GFP-positive S2 cells in S phase 200 min after heat shock expression of GFP, GFP-E2f1, GFP-E2f1^R161A^ or GFP-E2f1^PIP-3A^. After induction of GFP, all S phase cells in the population are GFP-positive (∼25%) because GFP protein is stable throughout the S2 cell cycle. In contrast, after induction of GFP-E2f1 expression, only ∼10% of GFP-positive cells are in S phase because GFP-E2f1 is targeted by CRL4^Cdt2^ for S phase destruction. The amount of GFP-positive cells in S phase after induction of GFP-E2f1^PIP3A^ or GFP-E2f1^R161A^ is equivalent to the amount after GFP induction, indicating that *Drosophila* E2f1 requires both a PIP box and a basic Arg residue 4 amino acids downstream of the PIP box for destruction during S phase. Here and in subsequent panels * indicates p<0.001 and error bars represent the standard error of at least three independent experiments. C) Third instar larval imaginal wing disc expressing RFP and GFP with en-Gal4. D-F) Flow cytometry profile of GFP expression versus DNA content from en-Gal4>GFP (D) en-Gal4>GFP-E2f1 (E) or en-GAL4>GFP-E2f1^Stable^ (F) trypsin-dissociated third instar imaginal wing disc cells. For each profile, data were acquired until 10,000 total cells were detected. The red boxes illustrate a representation of the S phase cells, and the blue dotted lines indicate the threshold for categorizing a cell as GFP positive (based on GFP negative control). Note that GFP expression (D) was higher than GFP-E2f1 or GFP-E2f1^Stable^ expression because the UASt promoter was used rather than UASp. G) Quantification by flow cytometry of GFP-positive S phase cells from trypsin-dissociated en-Gal4>GFP, GFP-E2f1, or GFP-E2f1^Stable^ wing discs. H) Quantification by flow cytometry of RFP-positive G1 phase cells from trypsin-dissociated, en-Gal4 wing discs expressing GFP or the indicated GFP-E2f1 fusion proteins. I) Quantification by flow cytometry of GFP-positive S2 cells in G1 phase after transient transfection of *Actin5C* promoter-driven constructs containing GFP or the indicated GFP-E2f1 fusion proteins that lack (grey) or contain (black) the PIP-3A mutation. J) Quantification by flow cytometry of GFP-positive S2 cells in S phase 200 min after heat shock expression of indicated GFP-E2f1 constructs that lack (grey) or contain (black) the PIP-3A mutation.
(TIF)

Figure S2 GMR-Gal4>GFP-E2F1 eye discs stained with anti-PH3. A) GMR>UAS-GFP eye disc. White box indicates example of areas shown in panel B and in Figure 3. Yellow box indicates areas shown in Figure 4. B) Detection of mitosis by anti-phospho histone H3 staining (red) of GMR-Gal4 third instar larval eye imaginal discs expressing GFP or the indicated GFP-E2f1 fusion proteins (green). Arrowheads indicate the position of the MF, with anterior to the left and posterior to the right. Bars = 5 µM.
(TIF)

Figure S3 E2f1^Stable^ does not induce apoptosis in G_1_ arrested embryonic cells. A–D) Stage 11 embryos (9–11 hours post egg laying) expressing GFP or the indicated GFP-E2f1 fusion proteins with en-Gal4. Green: GFP for transgene expression, Red: Cleaved Caspase-3, White: phospho-tyrosine for cell membrane marker, Blue: DAPI for nuclei). Epithelial cells (white) on the surface of the embryo have exited the cell cycle and are arrested in G_1_. E) En-Gal4>UAS-GFP embryo with a CC3-positive apoptotic cell (red) below the surface epithelial cells. This cell is most likely a neuronal cell and is shown as a positive control for CC3 detection. F) En-Gal4>UAS-*reaper* embryo shown to ensure that the epidermal cells respond to pro-apoptotic signals and accumulate CC3. Bars = 10 µm.
(TIF)

Figure S4 E2f1^Stable^ induces DNA damage in wing discs. A–C) Detection of DNA damage by anti-phospho-H2Av staining (red) in en-Gal4 third instar larval eye imaginal discs expressing GFP or the indicated GFP-E2f1 fusion proteins (green). D) Quantification of anti-phospho-H2Av staining. Foci above a calibrated threshold (ImageJ) were counted for each allele. n = 10 discs for each genotype. Both E2f1 alleles had significantly more foci than UAS-GFP alone (* = p<0.001).
(TIF)
