# Supplementary figures and images for: S Phase–Coupled E2f1 Destruction Ensures Homeostasis in Proliferating Tissues
Source: PLoS Genet. 2012 Aug 16;8(8):e1002831. doi: 10.1371/journal.pgen.1002831 (PMC3420931; doi:10.1371/journal.pgen.1002831)

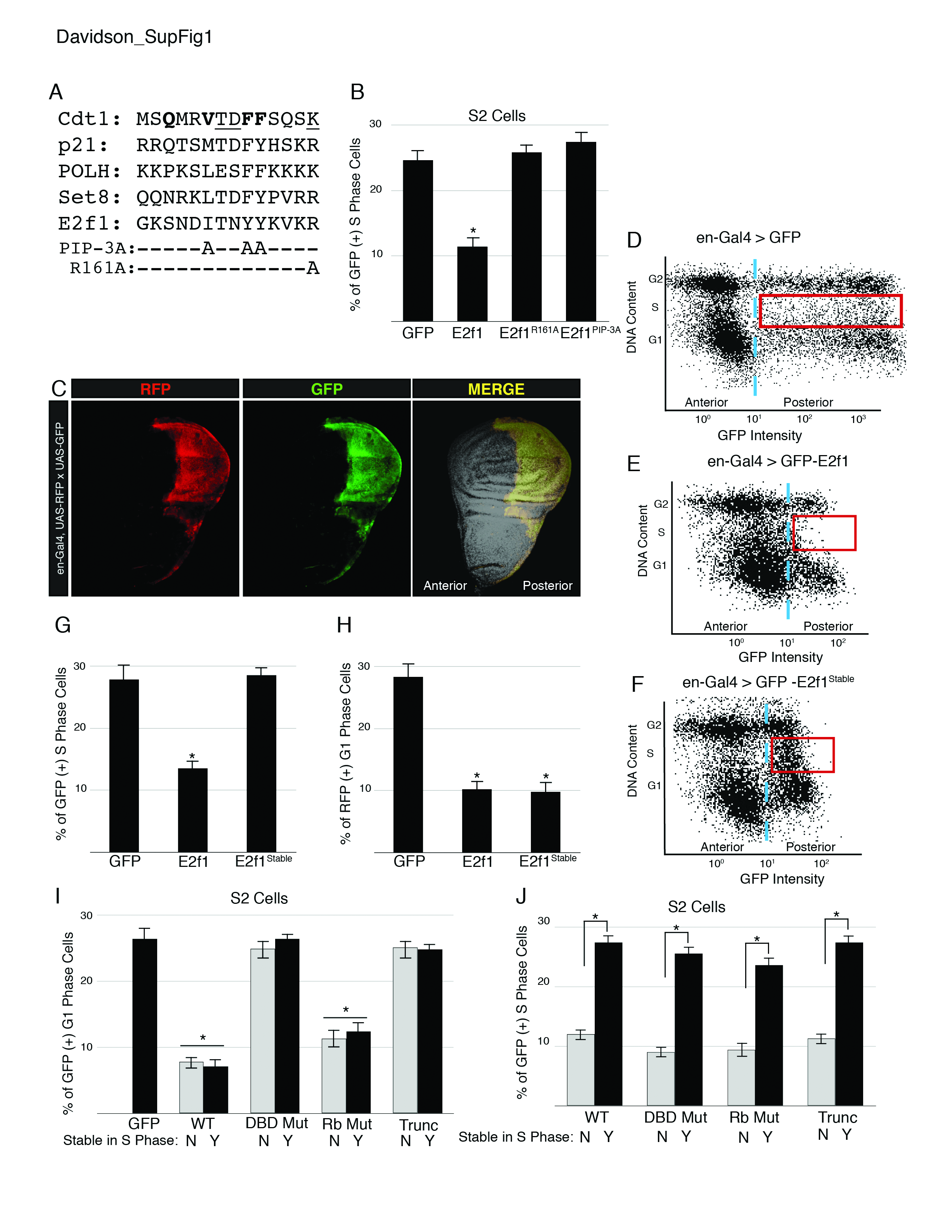

Supplement: Figure S1 — An in vivo assay for S phase-coupled E2f1 destruction. A) Alignment of PIP degrons from known CLR4Cdt2 substrates. Amino acids of the PIP box are bold and those of Cdt1 that interact with Cdt2 are underlined. E2f1 contains a PIP box located at amino acids 150–157. E2f1 also contains a basic Arg residue (R161) four amino acids downstream of the PIP box, much like the basic K+4 residue found in the Cdt1 PIP degron. Amino acid changes in E2f1PIP-3A and E2f1R161A mutants, which contain nonfunctional PIP degrons, are shown at the bottom. B) An S2 cell flow cytometry assay to quantify the number of GFP-positive cells that are in S phase. The graph indicates the percentage of GFP-positive S2 cells in S phase 200 min after heat shock expression of GFP, GFP-E2f1, GFP-E2f1R161A or GFP-E2f1PIP-3A. After induction of GFP, all S phase cells in the population are GFP-positive (∼25%) because GFP protein is stable throughout the S2 cell cycle. In contrast, after induction of GFP-E2f1 expression, only ∼10% of GFP-positive cells are in S phase because GFP-E2f1 is targeted by CRL4Cdt2 for S phase destruction. The amount of GFP-positive cells in S phase after induction of GFP-E2f1PIP3A or GFP-E2f1R161A is equivalent to the amount after GFP induction, indicating that Drosophila E2f1 requires both a PIP box and a basic Arg residue 4 amino acids downstream of the PIP box for destruction during S phase. Here and in subsequent panels * indicates p<0.001 and error bars represent the standard error of at least three independent experiments. C) Third instar larval imaginal wing disc expressing RFP and GFP with en-Gal4. D-F) Flow cytometry profile of GFP expression versus DNA content from en-Gal4>GFP (D) en-Gal4>GFP-E2f1 (E) or en-GAL4>GFP-E2f1Stable (F) trypsin-dissociated third instar imaginal wing disc cells. For each profile, data were acquired until 10,000 total cells were detected. The red boxes illustrate a representation of the S phase cells, and the blue dotted lines indicate the thres [file pgen.1002831.s001.tif]

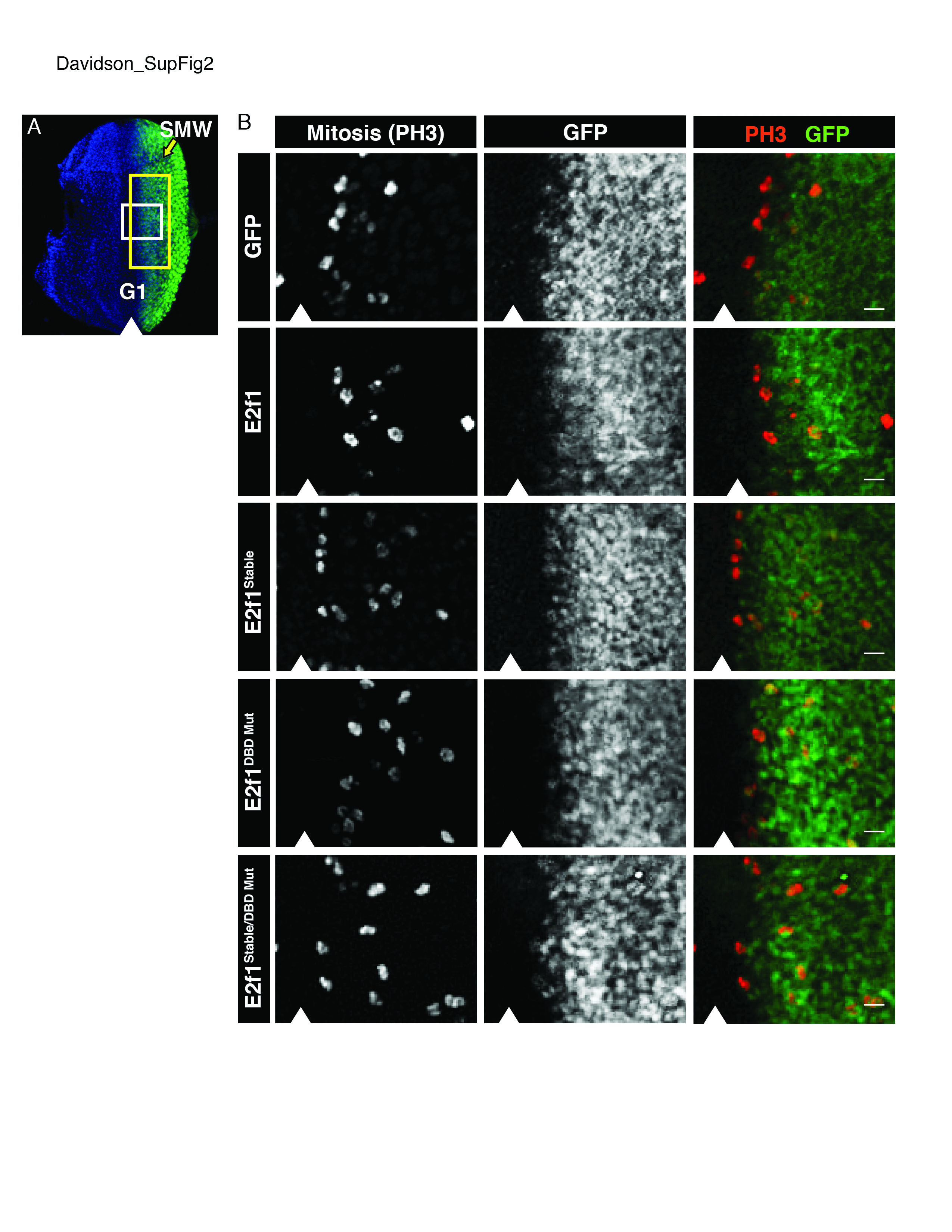

Supplement: Figure S2 — GMR-Gal4>GFP-E2F1 eye discs stained with anti-PH3. A) GMR>UAS-GFP eye disc. White box indicates example of areas shown in panel B and in Figure 3. Yellow box indicates areas shown in Figure 4. B) Detection of mitosis by anti-phospho histone H3 staining (red) of GMR-Gal4 third instar larval eye imaginal discs expressing GFP or the indicated GFP-E2f1 fusion proteins (green). Arrowheads indicate the position of the MF, with anterior to the left and posterior to the right. Bars = 5 µM. (TIF) [file pgen.1002831.s002.tif]

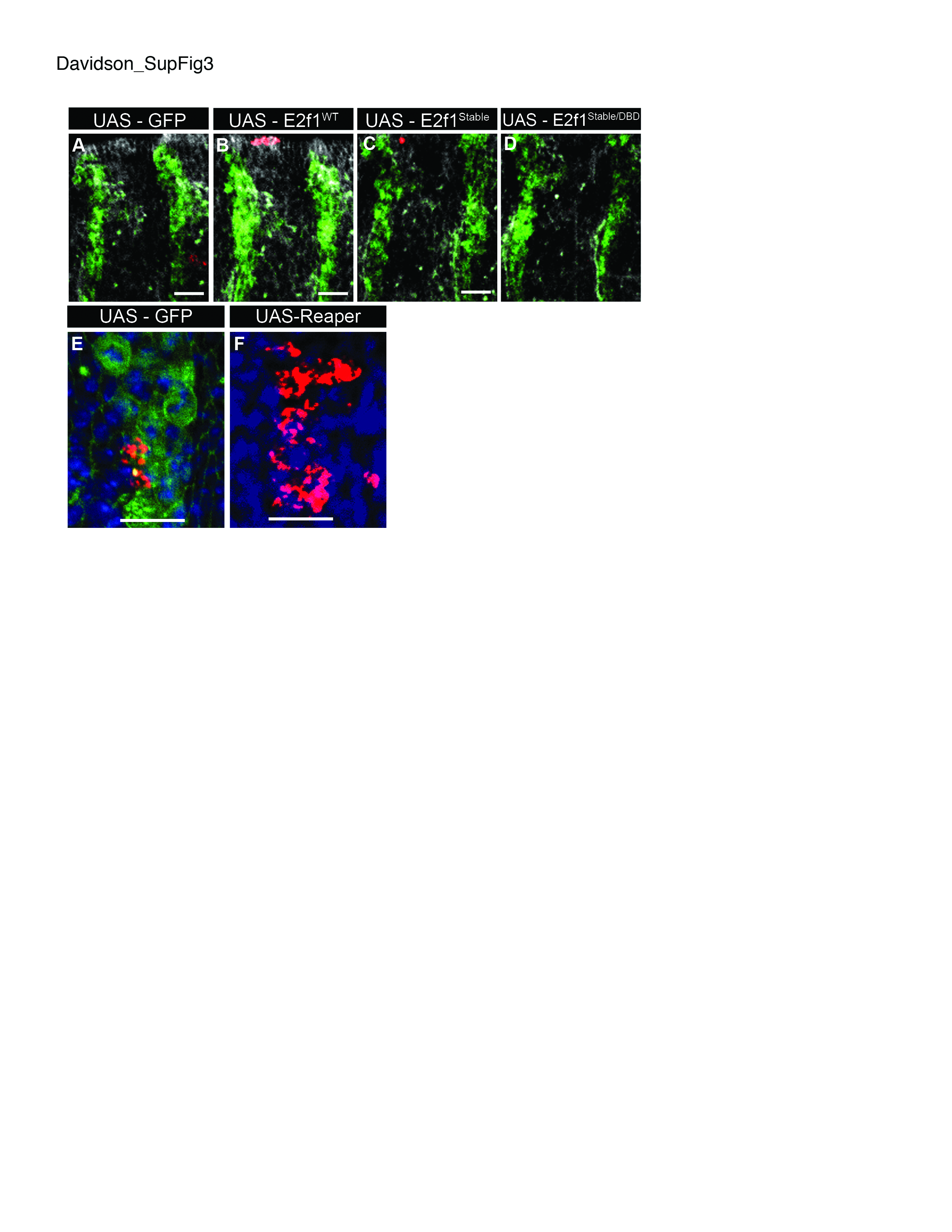

Supplement: Figure S3 — E2f1Stable does not induce apoptosis in G1 arrested embryonic cells. A–D) Stage 11 embryos (9–11 hours post egg laying) expressing GFP or the indicated GFP-E2f1 fusion proteins with en-Gal4. Green: GFP for transgene expression, Red: Cleaved Caspase-3, White: phospho-tyrosine for cell membrane marker, Blue: DAPI for nuclei). Epithelial cells (white) on the surface of the embryo have exited the cell cycle and are arrested in G1. E) En-Gal4>UAS-GFP embryo with a CC3-positive apoptotic cell (red) below the surface epithelial cells. This cell is most likely a neuronal cell and is shown as a positive control for CC3 detection. F) En-Gal4>UAS-reaper embryo shown to ensure that the epidermal cells respond to pro-apoptotic signals and accumulate CC3. Bars = 10 µm. (TIF) [file pgen.1002831.s003.tif]

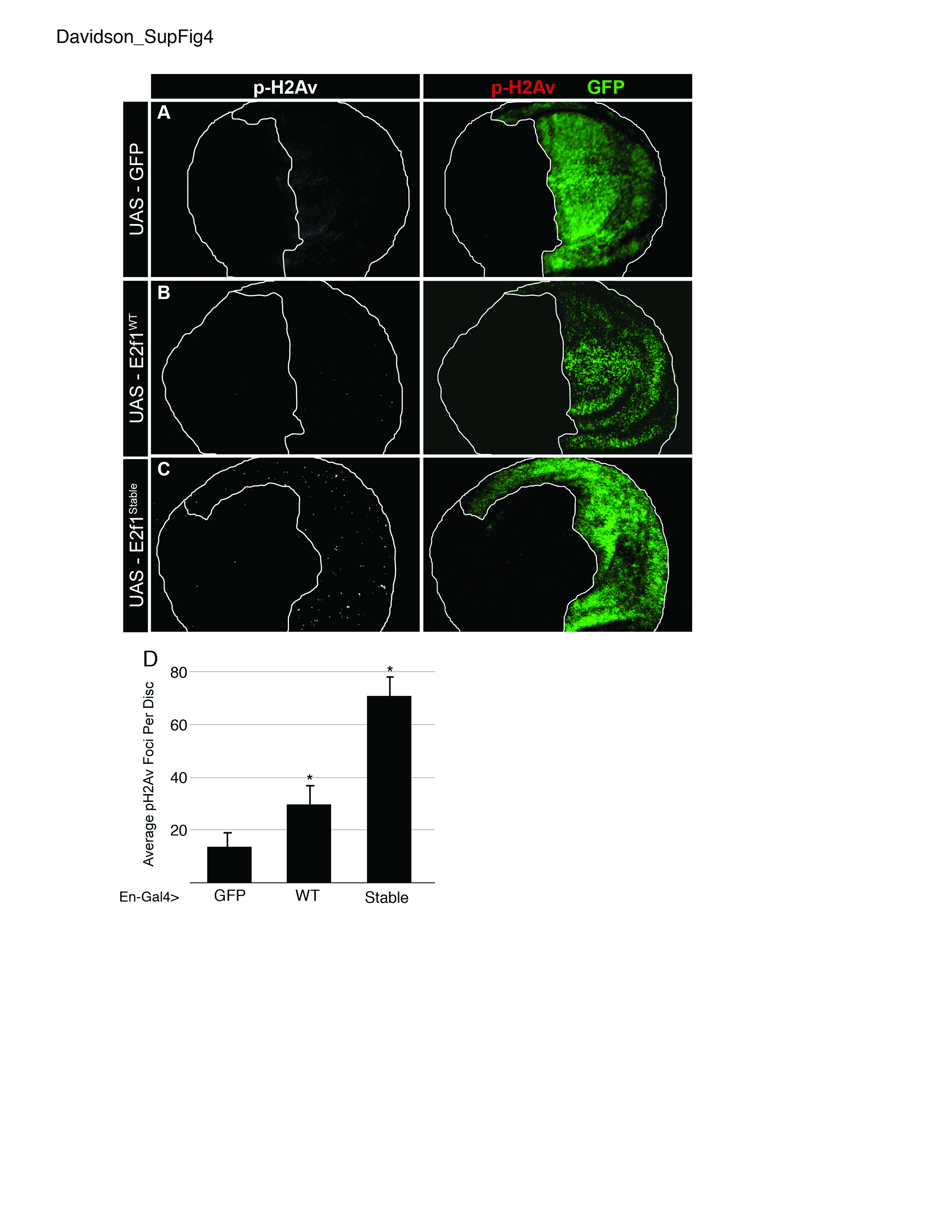

Supplement: Figure S4 — E2f1Stable induces DNA damage in wing discs. A–C) Detection of DNA damage by anti-phospho-H2Av staining (red) in en-Gal4 third instar larval eye imaginal discs expressing GFP or the indicated GFP-E2f1 fusion proteins (green). D) Quantification of anti-phospho-H2Av staining. Foci above a calibrated threshold (ImageJ) were counted for each allele. n = 10 discs for each genotype. Both E2f1 alleles had significantly more foci than UAS-GFP alone (* = p<0.001). (TIF) [file pgen.1002831.s004.tif]
